# A New Regression Model for the Prediction of the Stress–Strain Relations of Different Materials

**DOI:** 10.3390/ma16227145

**Published:** 2023-11-13

**Authors:** Yanli Lin, Yibo Su, Qilin Zhao, Shuo Wang, Hang Yuan, Xinyu Hu, Zhubin He

**Affiliations:** 1 School of Mechanical Engineering, Dalian University of Technology, Dalian 116024, China; linyanli@dlut.edu.cn (Y.L.); suyibo2021@163.com (Y.S.); m15034075046@163.com (Q.Z.); wshuo98@mail.dlut.edu.cn (S.W.); hangyuan@mail.dlut.edu.cn (H.Y.); hezb@dlut.edu.cn (Z.H.); 2 State Key Laboratory of High-Performance Precision Manufacturing, Dalian 116024, China

**Keywords:** experimental stress–strain relation, regression model, anisotropy, yielding behavior, plastic flow characteristic

## Abstract

Experimental flow stress–strain data under different stress states are often used to calibrate the plastic constitutive model of anisotropic metal materials or identify the appropriate model that is able to reproduce their plastic deformation behavior. Since the experimental stress–strain data are discrete, they need to be mathematically returned to a continuous function to be used to describe an equivalent hardening increment. However, the regression results obtained using existing regression models are not always accurate, especially for stress–strain curves under biaxial stress loading conditions. Therefore, a new regression model is proposed in this paper. The highest-order term in the recommended form of the new model is quadratic, so the functional relationships between stress–strain components can be organized into explicit expressions. All the experimental data of the uniform deformation stage can be substituted into the new model to reasonably reproduce the biaxial experimental stress–strain data. The regression results of experimental data show that the regression accuracy of the new model is greatly improved, and the residual square sum SSE of the regression curves of the new model reduced to less than 50% of the existing three models. The regression results of stress–strain curves show significant differences in describing the yield and plastic flow characteristics of anisotropic metal materials, indicating that accurate regression results are crucial for accurately describing the anisotropic yielding and plastic flow behaviors of anisotropic metal materials.

## 1. Introduction

The application of high-strength steel, aluminum alloy, magnesium alloy and titanium alloy materials has been increasing in various manufacturing industries, such as automobiles, aerospace, bicycles, etc. [1,2,3]. These metal materials generally exhibit significant anisotropy, including anisotropic yielding and anisotropic plastic flow behaviors [4,5,6]. Therefore, various experimental data from different directions and different stress states has been utilized to calibrate the plastic constitutive models of these anisotropic metal materials or identify the appropriate model which are able to reproduce the plastic deformation behavior of them [7,8,9].

Uniaxial tensile test results in three directions, namely the rolling direction (RD), transverse directions (TD) and the direction 45° from the RD of the material, respectively, and the flow stress–strain curves of biaxial tensile tests, plane–strain tension and disk compression experiments, etc., were commonly used to calibrate the plastic constitutive models of sheet metal or identify the proper model [7,10,11,12,13,14]. For instance, the plastic constitutive model of a 1.2 mm thick dual phase steel sheet with a tensile strength of 780 MPa was calibrated using the results of uniaxial tensions in RD, TD and 45° directions and equibiaxial tension [11], and its appropriateness was identified using the results of biaxial tensile tests that were performed using the cruciform specimen. The plastic constitutive model of an AA6022-T4 sheet with 1 mm thickness was calibrated via uniaxial tension, plane–strain tension and disk compression experiments [12]. Similarly, the plastic constitutive models of aluminum sheets of AA7075 with 1.5 mm thickness in two different tempers [13], a zinc-coated low carbon steel sheet (SPCD) with a thickness of 0.66 mm [14], a 590-MPa-class high-strength steel of 1.2 mm in thickness (590HSS) [15] were all determined and calibrated.

For anisotropic thin-walled metal tubes, the information of the flow stress–strain curves under different directions and different stress states are often provided via axial uniaxial tensile tests, hoop tensile tests [1,2], free bulging tests [16,17], biaxial controllable loading bulging tests [9,18,19], pure shearing tests [20], shear–tension tests [21,22], and so on. These flow stress–strain curves are often used to calibrate the plastic constitutive model of thin-walled metal, or to identify their aappropriateness.

When using experimental stress–strain data from different directions and different stress states to calibrate the plastic constitutive model of a material or determine its applicability, each set of experimental data must correspond to the same equivalent hardening state, that is, each set of data must correspond to the same level of plastic work [23,24]. Since the experimental stress–strain data are discrete, all of these experimental stress–strain data should be reproduced as continuous functions [25]. The regression precision will affect the specific expression of the plastic constitutive model, and furthermore will affect the theoretical prediction accuracy of the yielding and plastic flow behaviors of materials under complex stress states. Therefore, accurate regression of experimental data is crucial.

Nonlinear regression models have been widely used to describe the nonlinear behavior of materials. Currently, experimental stress–strain data are commonly reproduced using nonlinear regression models, such as power law, the second-order function model, the fourth-order function model, and so on [25,26,27,28,29,30,31]. But with the development of technology, many new materials have emerged, and many flow stress–strain data under biaxial stress state can be obtained using the new proposed test method. For the experimental stress–strain data of some new materials and some flow stress–strain data under a biaxial stress state, the regression accuracy of the existing model is not high enough [14]. Therefore, proposing new regression models for these experimental data will be a fundamental issue for improving the accuracy of plastic constitutive models.

In this paper, a new regression model is proposed, and analytical and numerical methods for determining the pending parameters of the new model are introduced. Then, the experimental flow stress–strain data of three kinds of thin-walled metal materials with obvious anisotropy are used to analyze the regression feasibility and universality of the new model. The regression results of other models were also analyzed for comparison. Finally, the effects of the regression results of different models on the yielding and plastic flow properties were analyzed through the experimental results of a 0.7 mm thick cold-rolled IF steel sheet. Consequently, it was found that the new regression model in the recommended form can well reproduce most of the experimental flow stress–strain data of the three kinds of thin-walled metal materials, whether it is in the uniaxial tensile stress state or in the biaxial stress state. The regression results of different models have a significant impact on the yielding and plastic flow properties of the material in the case of the 0.7 mm thick cold-rolled IF steel sheet.

## 2. The Expression and Analysis of the New Regression Model

In order to improve the regression accuracy of experimental stress–strain data, a new regression model is proposed.

The general expression of the new model is

(1) $$\sigma =\frac{a_{1}\varepsilon^{n}+a_{2}\varepsilon^{n-1}+\cdots +a_{n}\varepsilon +a_{n+1}}{\varepsilon^{m}+b_{1}\varepsilon^{m-1}+\cdots +b_{m-1}\varepsilon +b_{m}}$$

where, $a_{1},a_{2},\cdots ,a_{n},a_{n+1}$ and $b_{1},\cdots ,b_{m-1},b_{m}$ are the pending coefficients, *n* and *m* are nonnegative integers, n≥0, m≥0, *σ* is the flow stress, and *ε* is the plastic strain corresponding to *σ*.

*n* and *m* have different combinations. When *n* and *m* take different values, the forms of functions are different. They can be classified into four forms which are as follows. (1) Pure polynomial (i.e., the denominator is a constant), i.e., $b_{1},\cdots ,b_{m-1}=0$, $b_{m}$ is a constant; (2) the numerator is a constant, i.e., $a_{1},a_{2},\cdots ,a_{n}=0$, $a_{n+1}$ is a constant; (3) the order of the numerator is lower than the denominator’s, i.e., n≤m; and (4) the order of the numerator is higher than the denominator’s, i.e., m≤n, respectively.

The regression accuracy of the new general model is analyzed through regressing the typical biaxial experimental data $\sigma_{x}/\sigma_{y}=1:2$, and the regression results obtained through the four forms of the new general model are shown in Table A1 in the Appendix A. From Table A1, it can be seen that the accuracy of the corresponding regression curves is very good (R-square = 0.999, i.e., close to one, *RMSE* is less than 0.55, and *SSE* is no more than 5.51) when n and m take any combined data in Table A1 except for m = 2 or 3, n = 0 and n = 2, m = 0. This shows that the general model shown in Equation (1) is reasonable, and that proper combinations of m and n can be selected as needed. In addition, it can be seen that the difference between the regression results is very small when *m*, *n* ≥ 3. R-squares are all equal to 0.99, and SSEs are essentially the same.

**Figure 1:**
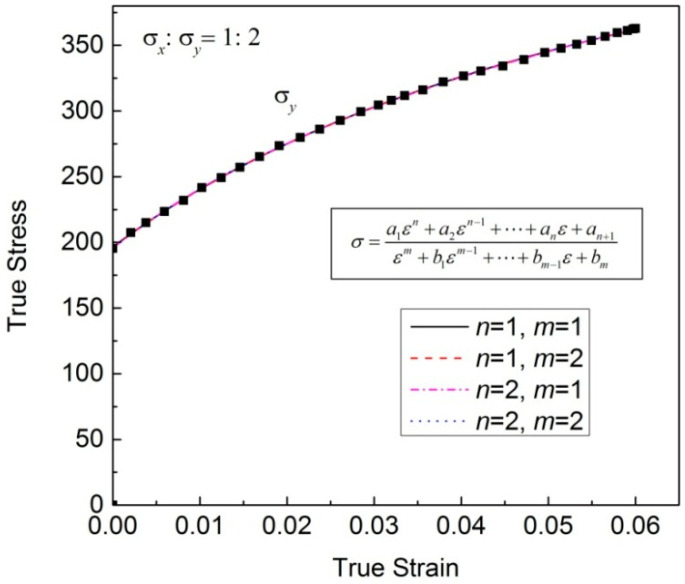
Regression results with the general model shown in Equation (1) when n,m=1,2.

The regression results when m and n are both less than 3 are shown in Figure 1. All the regression results are good, and the regression coefficients are shown in Table 1. The regression coefficients are too large for m = 2, n = 1 or 2; this is not conducive to the stability of the model, so it is not recommended to choose these two functions. Furthermore, from Table A1, it can be seen that the regression result of n = 2, m = 1 is more accurate than that of n =1, m = 1.

Therefore, after the comprehensive balance of regression accuracy, considering the monotonicity of the first derivative, whether it can be expressed as an explicit function of stress and strain and calculation efficiency, the combination of n = 2, m = 1 is first recommended. At this point, the corresponding specific expression of the new model is

(2) $$\sigma =\frac{a_{1}\varepsilon^{2}+a_{2}\varepsilon +a_{3}}{\varepsilon +b_{1}}$$

The new model shown in Equation (2) only has four undetermined coefficients and the highest-order term in the new model is quadratic, so the functional relationships between stress–strain components can be organized into explicit expressions. The analytic expression of the strain using the stress is given as follows:

(3) $$\varepsilon =\frac{\sigma -a_{2}+\sqrt{{\left(\sigma -a_{2}\right)}^{2}+4a_{1}\left(b_{1}\sigma -a_{3}\right)}}{2a_{1}}$$

Further, the new model shown in Equation (2) also has the advantage that the relationship between the stress increment dσ and the strain increment dε can be explicitly expressed alongside each other,

(4) $$d\sigma =\frac{a_{1}\varepsilon^{2}+2a_{1}b_{1}\varepsilon +a_{2}b_{1}-a_{3}}{{\left(b_{1}+\varepsilon \right)}^{2}}d\varepsilon$$

For a biaxial stress state, the equations of regression in both directions would be expressed as

(5) $$\left\{\begin{array}{l} d\sigma_{x}=\frac{a_{1x}\varepsilon_{x}^{2}+2a_{1x}b_{1x}\varepsilon_{x}+a_{2x}b_{1x}-a_{3x}}{{\left(b_{1x}+\varepsilon_{x}\right)}^{2}}d\varepsilon_{x}\;(a)\\ d\sigma_{y}=\frac{a_{1y}\varepsilon_{y}^{2}+2a_{1y}b_{1y}\varepsilon_{y}+a_{2y}b_{1y}-a_{3y}}{{\left(b_{1y}+\varepsilon_{y}\right)}^{2}}d\varepsilon_{y}\;(b) \end{array}\right.$$

where $d\sigma_{x}$ and $d\sigma_{y}$ are the stress increments in the direction *x* and direction *y*, respectively. $\varepsilon_{x}$ and $\varepsilon_{y}$ are the plastic strains in the direction *x* and direction *y*, respectively. $d\varepsilon_{x}$ and $d\varepsilon_{y}$ are the plastic strain increments in the direction *x* and direction *y*, respectively. $a_{1x}$, $a_{2x}$, $a_{3x}$ and $b_{1x}$ are the pending coefficients of the function in the direction *x*. $a_{1y}$, $a_{2y}$, $a_{3y}$ and $b_{1y}$ are the pending coefficients in the direction *y*.

Based on Equation (5), the expression of an equivalent increment on a biaxial stress state would be expressed as

(6) $$\left\{\begin{array}{c} d^{2}w^{p}=f\left(a_{1x},a_{2x},a_{3x},b_{1x},a_{1y},a_{2y},a_{3y},b_{1y},\alpha ,d\varepsilon_{x}\right)=\left(\frac{A}{B^{2}}+\frac{1}{\alpha^{2}}\frac{A^{2}C^{2}}{B^{4}D}\right){\left(d\varepsilon_{x}\right)}^{2}\\ A=a_{1x}\varepsilon_{x}^{2}+2a_{1x}b_{1x}\varepsilon_{x}+a_{2x}b_{1x}-a_{3x};\\ B=\left(b_{1x}+\varepsilon_{x}\right);\\ C=b_{1y}+\varepsilon_{y};\\ D=a_{1y}\varepsilon_{y}^{2}+2a_{1y}b_{1y}\varepsilon_{y}+a_{2y}b_{1y}-a_{3y};\\ \varepsilon_{y}=\frac{\sigma_{x}/\alpha -a_{2y}+\sqrt{{\left(\sigma_{x}/\alpha -a_{2y}\right)}^{2}+4a_{1y}\left(b_{1y}\sigma_{x}/\alpha -a_{3y}\right)}}{2a_{1y}} \end{array}\right.$$

where $d^{2}w^{p}$ is the second-order differentiation of plastic work, *α* is the stress ratio, and $\alpha =d\sigma_{x}/d\sigma_{y}$.

If $d\varepsilon_{x}$ is chosen as the increment step, $d\varepsilon_{x}$ can be calculated using the $\varepsilon_{x}$, $\varepsilon_{y}$, $\sigma_{x}$ and *α* of the current step through Equation (6). Further, $d\sigma_{x}$, $d\sigma_{y}$ and $d\varepsilon_{y}$ can be calculated and determined sequentially using Equation (5a), stress ratio *α*, and Equation (5b). So far, all the stress–strain components in the next step of the two directions under biaxial stress state have been obtained.

### 2.1. Analytical Method for Determining the Pending Parameters of the New Model

For the new model shown in Equation (2), the pending coefficients *a*_1_, *a*_2_, *a*_3_ and *b*_1_ can be determined using four different sets of experimental data. If the experimental data $\left(\varepsilon_{i},\sigma_{i}\right)$ (where, *i* = 1, 2, 3, 4) are known, then

(7) $$\sigma_{i}=\frac{a_{1}\varepsilon_{i}^{2}+a_{2}\varepsilon_{i}+a_{3}}{\varepsilon_{i}+b_{1}}$$

Further, Equation (7) can be given as

(8) $$a_{1}\varepsilon_{i}^{2}+a_{2}\varepsilon_{i}+a_{3}-b_{1}\sigma_{i}=\sigma_{i}\varepsilon_{i}$$

Substituting four known sets of experimental data, Equation (8) can be expressed in a matrix form:

(9) $$\left[\begin{array}{cccc} \varepsilon_{1}^{2} & \varepsilon_{1} & 1 & -\sigma_{1}\\ \varepsilon_{2}^{2} & \varepsilon_{2} & 1 & -\sigma_{2}\\ \varepsilon_{3}^{2} & \varepsilon_{3} & 1 & -\sigma_{3}\\ \varepsilon_{4}^{2} & \varepsilon_{4} & 1 & -\sigma_{4} \end{array}\right]\left[\begin{array}{c} a_{1}\\ a_{2}\\ a_{3}\\ b_{1} \end{array}\right]=\left[\begin{array}{c} \sigma_{1}\varepsilon_{1}\\ \sigma_{2}\varepsilon_{2}\\ \sigma_{3}\varepsilon_{3}\\ \sigma_{4}\varepsilon_{4} \end{array}\right]$$

Defining the pending coefficients vector as $c_{s}={\left[\begin{array}{cccc} a_{1} & a_{2} & a_{3} & b_{1} \end{array}\right]}^{T}$, Equation (9) can be given as

(10) $$A_{s}c_{s}=B_{s}$$

where $A_{s}=\left[\begin{array}{cccc} \varepsilon_{1}^{2} & \varepsilon_{1} & 1 & -\sigma_{1}\\ \varepsilon_{2}^{2} & \varepsilon_{2} & 1 & -\sigma_{2}\\ \varepsilon_{3}^{2} & \varepsilon_{3} & 1 & -\sigma_{3}\\ \varepsilon_{4}^{2} & \varepsilon_{4} & 1 & -\sigma_{4} \end{array}\right]$, is a matrix of 4×4, and $B_{s}=\left[\begin{array}{c} \sigma_{1}\varepsilon_{1}\\ \sigma_{2}\varepsilon_{2}\\ \sigma_{3}\varepsilon_{3}\\ \sigma_{4}\varepsilon_{4} \end{array}\right]$ is a 4-dimensional column vector.

If matrix A is reversible, i.e., $\det\left(A\right)\neq 0$, the pending coefficients of the model can be obtained as follows:

(11) $$c_{s}={\left(A_{s}\right)}^{-1}B_{s}$$

For the general expression of the new model shown in Equation (1), the pending coefficients $a_{1},a_{2},\cdots ,a_{n+1}$ and $b_{1},b_{2},\cdots ,b_{m}$ can be determined using n+m+1 different sets of experimental data. Suppose that the experimental data $\left(\varepsilon_{i},\sigma_{i}\right)$ (where i=1, 2,⋯,n+m+1) are known, the pending coefficient vector $P={\left[a_{1}\;\cdots \;a_{n+1}\;b_{1}\;\cdots \;b_{m}\right]}^{T}$ can be expressed as

(12) $$P={\left[\begin{array}{cccccc} a_{1} & \cdots & a_{n+1} & b_{1} & \cdots & b_{m} \end{array}\right]}^{T}=A^{-1}B$$

where $A=\left[\begin{array}{cccccc} \varepsilon_{1}^{n} & \cdots & 1 & -\sigma_{1}\varepsilon_{1}^{m-1} & \cdots & -\sigma_{1}\\ \vdots & \vdots & \vdots & \vdots & \vdots & \vdots \\ \varepsilon_{n+1}^{n} & \cdots & 1 & -\sigma_{n+1}\varepsilon_{n+1}^{m-1} & \cdots & -\sigma_{n+1}\\ \varepsilon_{n+2}^{n} & \cdots & 1 & -\sigma_{n+2}\varepsilon_{n+2}^{m-1} & \cdots & -\sigma_{n+2}\\ \vdots & \vdots & \vdots & \vdots & \vdots & \vdots \\ \varepsilon_{n+m+1}^{n} & \cdots & 1 & -\sigma_{n+m+1}\varepsilon_{n+m+1}^{m-1} & \cdots & -\sigma_{n+m+1} \end{array}\right]$ is a matrix of $\left(n+m+1\right)\times \left(n+m+1\right)$, and $B=\left[\begin{array}{c} \sigma_{1}\varepsilon_{1}^{m}\\ \vdots \\ \sigma_{n+1}\varepsilon_{n+1}^{m}\\ \sigma_{n+2}\varepsilon_{n+2}^{m}\\ \vdots \\ \sigma_{n+m+1}\varepsilon_{n+m+1}^{m} \end{array}\right]$ is a $\left(n+m+1\right)$-dimensional column vector.

### 2.2. Numerical Method for Determining the Pending Parameters of the New Model

As we all know, there are always some test errors in experimental data due to reasons such as test equipment, experimental conditions, and experimental environment. The experimental flow stress–strain curve is always fluctuating throughout the deformation process, especially for biaxial experimental stress–strain curves under controllable loading paths. As a feedback control is required, the fluctuation is bigger. Therefore, if analytical methods are used to determine the undetermined coefficients of the new model, it means that only four experimental points are used to determine the entire regression curve, and the regression accuracy will inevitably be affected by the selected points. Thus, the least-squares algorithm is recommended for substituting all the experimental data points in the plastic deformation stage into the expression of the new model to determine the pending coefficients, in order to minimize the impact of test errors on the regression results and obtain more accurate regression results.

The general model shown in Equation (1) can be represented as

(13) $$\sigma =f\left(\varepsilon ,P\right)$$

where $P={\left[a_{1}\;\cdots \;a_{n+1}\;b_{1}\;\cdots \;b_{m}\right]}^{T}$ is the pending coefficient vector. If there are n+m+1 different sets of experimental data $\left(\varepsilon_{i},\sigma_{i}\right)$ (where i=0,1,……,n+m+1) known, the optimum parameter vector P will be determined through minimizing the sum of squared residuals [32], i.e.,

(14) $$P=\arg\min_{P}\left\{M\left(P\right)\right\}$$

$M\left(P\right)$ is the objective function for solving the pending coefficient vector. Usually, the sum of squared residuals is chosen as the objective function:

(15) $$M\left(P\right)=\sum_{i=1}^{n+m+1}{\left(f\left(\varepsilon_{i},P\right)-\sigma_{i}\right)}^{2}=g^{T}\left(P\right)g\left(P\right)$$

where $g(P)={\left[g_{1}(P),g_{2}(P),\cdots ,g_{N}(P)\right]}^{T}$ is the residual vector under the given experimental data, and *N* is a positive integer.

The Levenberg–Marquardt [33,34] algorithm improves the iteration accuracy and stability by introducing a damping term, so it is chosen to solve the parameter vector P, which can be expressed as

(16) $$P^{k+1}=P^{k}-{\left[J^{T}\left(P^{k}\right)J\left(P^{k}\right)+\mu I\right]}^{-1}J^{T}\left(P^{k}\right)g\left(P^{k}\right)$$

where μ is the damping constant which is a positive constant, and I is an $m_{1}\times m_{1}$ identity matrix. $P^{k+1}$ and $P^{k}$ are the iterative values of the parameter vector in steps k+1 and k, respectively, $g(P^{k})$ is the residual vector of step k, and $J(P^{k})$ is a Jacobin matrix of step k, which can be expressed as

(17) $$J\left(P^{k}\right)={\left[\begin{array}{cccc} \frac{\partial g_{1}\left(P\right)}{\partial a_{1}} & \frac{\partial g_{1}\left(P\right)}{\partial a_{2}} & \cdots & \frac{\partial g_{1}\left(P\right)}{\partial b_{m}}\\ \frac{\partial g_{2}\left(P\right)}{\partial a_{1}} & \frac{\partial g_{2}\left(P\right)}{\partial a_{2}} & \cdots & \frac{\partial g_{2}\left(P\right)}{\partial b_{m}}\\ \vdots & \vdots & & \vdots \\ \frac{\partial g_{N}\left(P\right)}{\partial a_{1}} & \frac{\partial g_{N}\left(P\right)}{\partial a_{2}} & \cdots & \frac{\partial g_{N}\left(P\right)}{\partial b_{m}} \end{array}\right]}_{P=P^{k}}$$

When the value of μ is small enough, LM algorithm is similar to the Gauss–Newton algorithm. When μ is big enough, the LM algorithm is similar to the gradient descent method. The constant μ can be self-adaptively adjusted in real time; therefore, the LM algorithm has the advantages of both the Gauss–Newton algorithm and the gradient descent method. In addition, $J^{T}\left(c^{k}\right)J\left(c^{k}\right)+\mu I$ is an invertible matrix, so the stability of the algorithm can be guaranteed.

The specific steps to solve the pending coefficient vector P using the LM algorithm are as follows:

Step 1 Determine the value of the constant matrix η, which is used to control the convergence error; the initial value $\mu_{0}$; the scaling factor λ, which is used to adjust the damping constant; and the initial value of pending coefficient vector $P^{0}$.

Step 2 Calculate $g(P^{k})$, $J\left(P^{k}\right)$ and $F\left(P^{k}\right)$.

Step 3 Calculate the iterative step.

Step 4 Calculate $P^{k+1}=P^{k}+h^{k}$ and $F\left(P^{k+1}\right)$.

Step 5 if $F\left(P^{k+1}\right)<F\left(P^{k}\right)$, then $\mu_{k+1}=\frac{\mu_{k}}{\lambda }$.

Step 6 if $F\left(P^{k+1}\right)\geq F\left(P^{k}\right)$, then $\mu_{k+1}=\lambda \mu_{k}$.

Step 7 if $\left\|h^{k}\right\|\leq \eta$, the iteration is terminated, and $P^{k+1}$ is the result. Otherwise, let k=k+1, and go to step 2.

## 3. Research Program

In order to verify the following advantages of the new model in regressing the flow stress–strain data of metal materials with different stress states and properties (such as regression feasibility and universality, regression accuracy, and stability), as well as the effects of the regression results on the yielding property and plastic flow behavior, the following research program is proposed.

Experimental flow stress–strain data of three kinds of materials with obvious anisotropy were used to verify regression feasibility and universality in this paper. The first kind of experimental data used in this study are from the results of the biaxial tensile test using cruciform specimens of 0.7 mm thick cold-rolled IF steel sheets, involving the data of the stress states σ*_x_*/σ*_y_* = 4:1, 2:1, 4:3, 1:4, 1:2, 3:4, 1:1 and 1:0 [7]. The *r* values at 0°, 45° and 90° (transverse direction; TD) to the rolling direction (RD) are 2.27, 1.77 and 2.65, respectively. The RD and TD directions of the material are defined as the x- and y-axes, respectively. The second kind of experimental data are from the results of controlled hydro-bulging tests of 1.9 mm thick extruded 6061F aluminum alloy tubes with a 40 mm initial outer diameter, including the data of the stress states $\sigma_{z}/\sigma_{\theta }=6:8$, 8:7 and −4:8 [28], where $\sigma_{z}$ and $\sigma_{\theta }$ are the axial and circumferential stresses, respectively. The last kind of experimental data are from the results of uniaxial tensile test in different directions of hot-rolled magnesium alloy sheet [35].

In order to illustrate the feasibility and superiority of the new model, the regression results of it were compared with the results reproduced by the second-order function model (abbreviated as SOF model) and power functions. The expression of the SOF model is

(18) $$\begin{array}{l} X_{1}{\left(\sigma_{\max}-\sigma \right)}^{2}+X_{2}\left(\varepsilon -\varepsilon_{y}\right)\left(\sigma_{\max}-\sigma \right)+X_{3}{\left(\varepsilon -\varepsilon_{y}\right)}^{2}-1=0\\ \left\{\begin{array}{l} X_{1}=\frac{1}{{\left(\sigma_{\max}-\sigma_{y}\right)}^{2}},\;X_{3}=\frac{1}{{\left(\varepsilon_{\max}-\varepsilon_{y}\right)}^{2}}\\ X_{2}=\left[1-{\left(\frac{\varepsilon_{A}-\varepsilon_{y}}{\varepsilon_{\max}-\varepsilon_{y}}\right)}^{2}-{\left(\frac{\sigma_{\max}-\sigma_{A}}{\sigma_{\max}-\sigma_{y}}\right)}^{2}\right]\frac{1}{\left(\sigma_{\max}-\sigma_{A}\right)\left(\varepsilon_{A}-\varepsilon_{y}\right)} \end{array}\right. \end{array}$$

where $\sigma_{y}$ and $\varepsilon_{y}$ are the initial yielding stress and the corresponding strain, $\sigma_{\max}$ and $\varepsilon_{\max}$ are the maximum stress and the corresponding strain, $\varepsilon_{A}$ and $\sigma_{A}$ are the strain and stress of experimental point A, which is located between the initial yield point and the maximum stress point, such as the 5% strain point, or some other point with a different strain value, and $X_{1},X_{2},X_{3}$ are the three pending coefficients used to address the experimental data.

The power function is

(19) $$\sigma =K\varepsilon^{n}$$

where *K* is the strength coefficient, and *n* is the strain hardening exponent.

The regression results of the new model were compared with results of the SOF model and power function. The regression accuracy of these models was evaluated using the residual sum of squares *SSE* ($SSE=\sum {\left(\sigma_{theoretical}-\sigma_{\mathrm{experimental}}\right)}^{2}$), the mean absolute percentage error *MAPE* ($MAPE=\sum \left(\left|\sigma_{theoretical}-\sigma_{\mathrm{experimental}}\right|\cdot 100/\sigma_{\mathrm{experimental}}\right)/n_{*}$) (where $n_{*}$ is the number of samples, and $n_{*}=1$ for this study), and residuals of each point δ ($\delta =\left(\sigma_{theoretical}-\sigma_{\mathrm{experimental}}\right)$) from overall and local angles, respectively. The regression results of the SOF model obtained separately with three different intermediate locations of point A were analyzed due to the sensitivity of the point A used for the regression [30]. The point A is located at $\varepsilon_{A}=1/4\varepsilon_{{}_{\max}}$, $\varepsilon_{A}=1/2\varepsilon_{{}_{\max}}$ and $\varepsilon_{A}=3/4\varepsilon_{{}_{\max}}$, respectively.

Finally, the experimental results of the 0.7 mm thick cold-rolled IF steel sheet were used to evaluate the effects of regression accuracy on the yielding and plastic flow properties of the material.

## 4. Results and Discussion

### 4.1. Regression Results of the New Model and Other Models

The regression universality, regression accuracy, and stability of the new model were discussed using the experimental flow stress–strain data of different stress states and material properties.

#### 4.1.1. Comparative Analysis of Regression Results of Different Models

**Figure 2:**
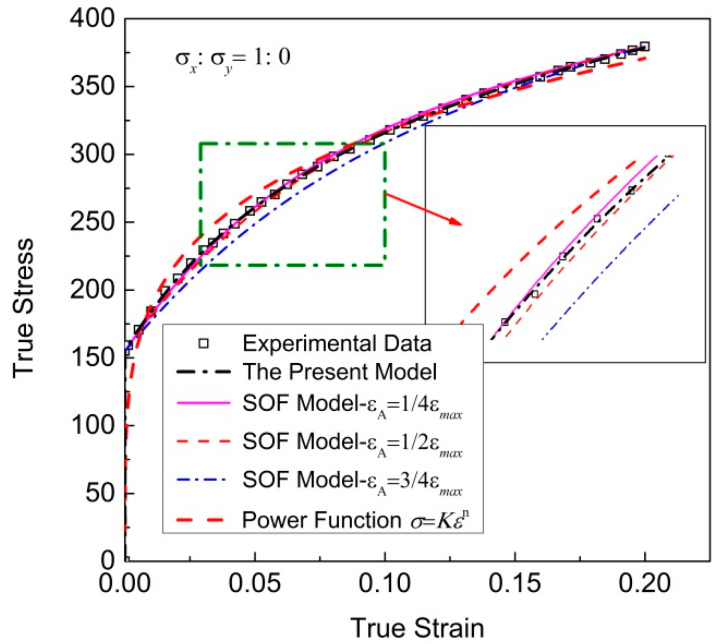
Regression results of cold-rolled IF steel sheets with different models, and the corresponding discrete experimental data.

The regression results of different models are comparatively analyzed through the experimental data of the uniaxial tensile stress state. Figure 2 shows the regression results of the cold-rolled IF steel sheet with different models and the corresponding discrete experimental data. The regression parameters of these models are shown in Table 2 and Table 3.

It can be seen that the regression result of the new model can well reproduce the experimental data, which are significantly better than those of other models. From Table 3, it can be seen that the three coefficients of the SOF model differ greatly, and *X*_1_ is too small. Table 4 shows the overall *error SSE*s *and MAPE* of regression results using the new model; the SOF model with *ε*_A_ = 1/4*ε*_max_, *ε*_A_ = 1/2*ε*_max_ and *ε*_A_ = 3/4*ε*_max_; and the power function. Regardless of *SSE* or *MAPE*, the result of the new model is the smallest, closer to the ideal case of *SSE* = 0, followed by the SOF model, with *ε*_A_ = 1/4*ε*_max_; the other two errors corresponding to the SOF model with *ε*_A_ = 1/2*ε*_max_ and *ε*_A_ = 3/4*ε*_max_ are much larger. The *SSE* of the regression result obtained using the power function is the maximum. The *SSE* of the new model is only 6.5% of the SOF model with *ε*_A_ = 1/4*ε*_max_. The predicting accuracy of the new model is better than that of the SOF model, and much better than the power function. In addition, the highest-order term in the new model is quadratic, so the functional relationships between stress–strain components can be easily organized into explicit expressions.

**Figure 3:**
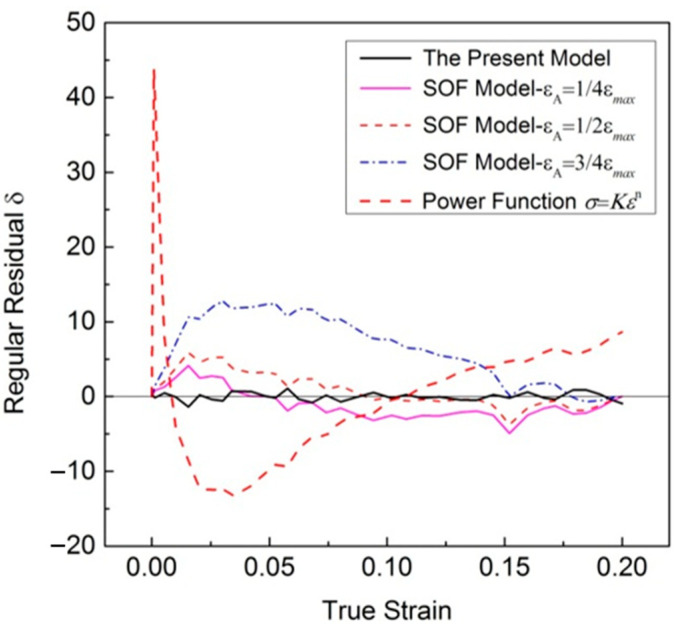
Total fitting deviation of uniaxial tensile stress–strain relationships.

Figure 3 shows the total fitting deviation dealing with uniaxial tensile stress–strain relationships. The maximum regular residual δ of the new model is 1.38 MPa, while most of regular residual δ obtained using the SOF model and power function is greater than the value. It is obvious that better results can be obtained with the new model.

Furthermore, the results in Table 3 and Table 4 show that the regression results of the SOF model were significantly affected by the value of point A. Among the three selected point A, the *SSE* is the smallest when *ε*_A_ = 1/4*ε*_max_, and the biggest when *ε*_A_ = 3/4*ε*_max_.

#### 4.1.2. Regression Results of Biaxial Stress State

**Figure 4:**
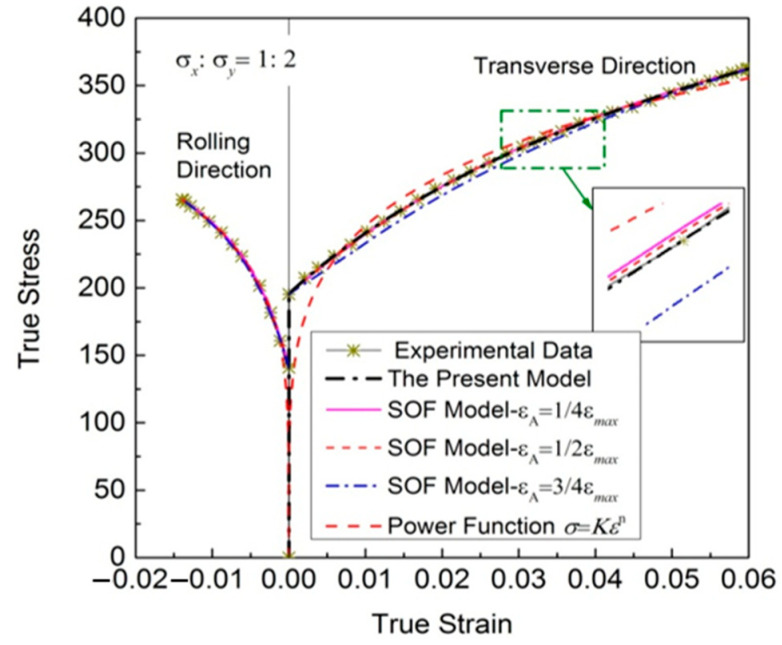
Experimental stress–strain relationships of a cold-rolled IF steel sheet with a stress ratio of $\sigma_{x}/\sigma_{y}=1:2$, reproduced with different functions.

Figure 4 shows the regression results of stress ratio $\sigma_{x}/\sigma_{y}=1:2$ of cold-rolled IF steel sheets with different models, and the corresponding discrete experimental data. The corresponding regression parameters of these models are shown in Table 5 and Table 6. From Table 6, it also can be seen that *X*_1_ is too small, and *X*_1_ and *X*_3_ vary widely, which is not conducive to the stable calculation of the model. $\varepsilon_{A}$ has a significant effect on the coefficient *X*_2_, which will lead to different regression results.

From Figure 4, it also can be seen that the regression accuracy of the SOF model is sensitive to the point A, which is an unstable regression model. Different points A are selected, the results vary widely. When the selected point A is unreasonable, the regression accuracy decreases significantly, and the SSE rises sharply. The regression results obtained using the power function significantly deviate from the experimental data. Moreover, there are no longer physical meanings to the regression results of many biaxial flow stress–strain data using power law, unlike the regression results of uniaxial tensile stress–strain data.

Table 7 shows the overall error SSEs and MAPE of the new model, the SOF model with $\varepsilon_{A}=1/4\varepsilon_{{}_{\max}}$, $\varepsilon_{A}=1/2\varepsilon_{{}_{\max}}$ and $\varepsilon_{A}=3/4\varepsilon_{{}_{\max}}$, and the power function along direction *y*. For the biaxial stress state, regardless of *SSE* or *MAPE*, the result of the new model is still the smallest, followed by the SOF model with $\varepsilon_{A}=1/2\varepsilon_{{}_{\max}}$, which has a significant regression advantage. Furthermore, all the experimental points in the plastic deformation stage can be used to optimize the regression results of experimental curves through this new model, using numerical algorithms; thus, the experimental data are evenly distributed on both sides of the regression curve, as well as the calculation error of the plastic work up and down offset. The regression results are reasonable and stable.

**Figure 5:**
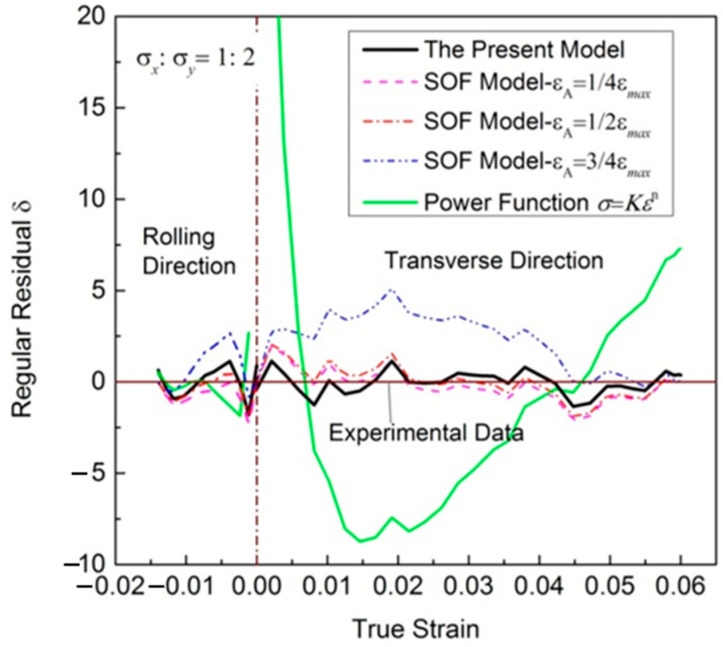
Total fitting deviation of experimental stress–strain relationships with stress ratio $\sigma_{x}/\sigma_{y}=1:2$.

Figure 5 presents the results of the deviation analysis. The residuals reproduced by the new model in the whole plastic deformation process are evenly distributed on both sides, with a maximum stress deviation of no more than 2.5 MPa. The predicting accuracy of this suggested model looks better than that of the SOF model, and much better than that of the power function, while the regular residuals reproduced by the SOF model with $\varepsilon_{A}=1/4\varepsilon_{{}_{\max}}$, $\varepsilon_{A}=1/2\varepsilon_{{}_{\max}}$ and $\varepsilon_{A}=3/4\varepsilon_{{}_{\max}}$ are different. Compared with the other two values, the residual distribution is more uniform when $\varepsilon_{A}=1/2\varepsilon_{{}_{\max}}$. The regression residuals are biased to the positive side when $\varepsilon_{A}=3/4\varepsilon_{{}_{\max}}$; the regression curve is always lower than the experimental results.

**Figure 6:**
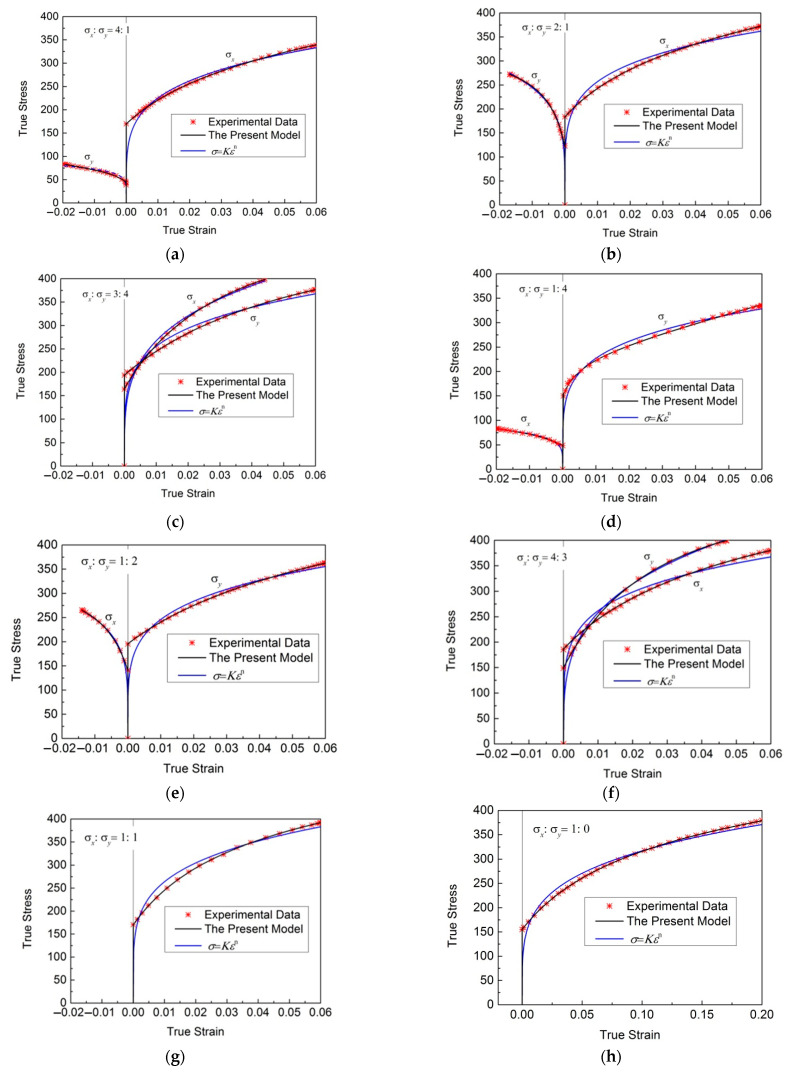
Regression results of various stress ratios of cold-rolled IF steel sheets with different models and the corresponding discrete experimental data. (**a**) $\sigma_{x}:\sigma_{y}=4:1$; (**b**) $\sigma_{x}:\sigma_{y}=2:1$; (**c**) $\sigma_{x}:\sigma_{y}=3:4$; (**d**) $\sigma_{x}:\sigma_{y}=1:4$; (**e**) $\sigma_{x}:\sigma_{y}=1:2$; (**f**) $\sigma_{x}:\sigma_{y}=4:3$; (**g**) $\sigma_{x}:\sigma_{y}=1:1$; (**h**) $\sigma_{x}:\sigma_{y}=1:0$.

Figure 6 shows the regression results of various stress ratios of cold-rolled IF steel sheets with different models and the corresponding discrete experimental data. The matched regression parameters of these models are shown in Table 8. From Figure 6, it can be seen that there are significant differences in the experimental flow stress–strain curves under different stress states. However, the regression results of the new model all well reproduce the experimental data of these stress ratios. The *SSE*s of the new model and the power function are shown in Table 2 and Table 3. Table 2 shows the *SSEs* in the direction *x,* and Table 3 shows the *SSEs* in the direction y. Table 9 shows the *SSE* of the new model is only $10^{-5}∼10^{-6}$ times that of the power function, and the regression results of the biaxial stress states have been significantly improved.

The regression feasibility and universality of these models are briefly analyzed using the following experimental flow stress–strain data, including the experimental data of an extruded 6061F aluminum alloy tube obtained through controlled hydro-bulging tests, and the experimental data of hot-rolled magnesium alloy sheet measured via the uniaxial tension in different directions.

**Figure 7:**
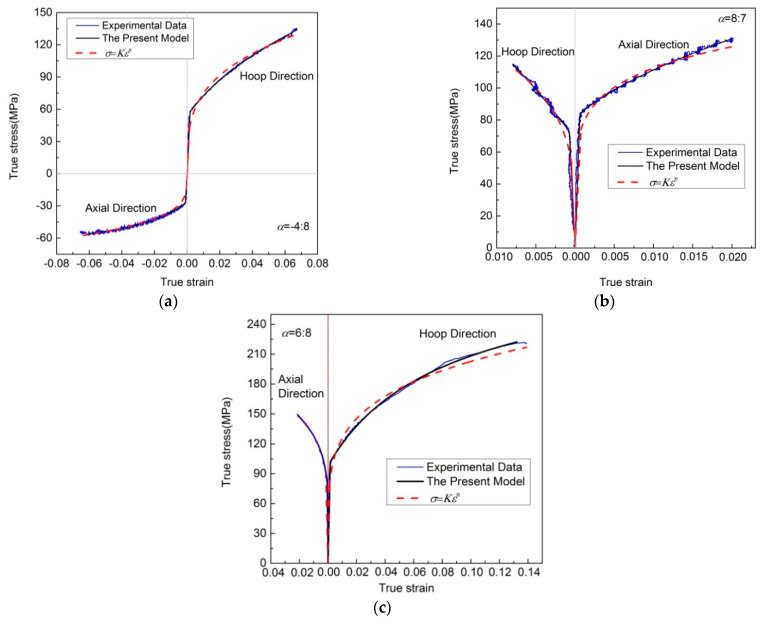
Experimental flow stress–strain curves of extruded 6061F aluminum alloy tubes measured via controlled hydro-bulging tests and the regression results of different models. (**a**) α=4:8; (**b**) α=8:7; (**c**) α=6:8.

Figure 7 displays the experimental flow stress–strain curves of an extruded 6061F aluminum alloy tube with stress ratios −4:8, 6:8 and 8:7 [28] measured through controlled hydro-bulging tests and the regression results of different models. It can be seen that there are significant differences in the experimental flow stress–strain curves under different stress states. However, they all can be well reproduced, using a numerical algorithm, through the new model.

**Figure 8:**
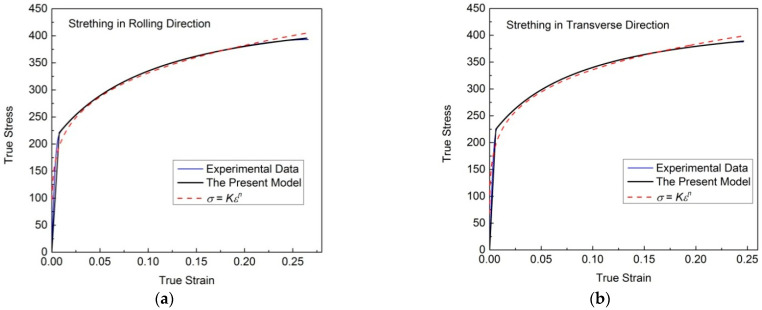
Uniaxial tensile stress–strain curves of hot-rolled magnesium alloy sheets in different directions, and the corresponding results reproduced by different models. (**a**) Strething in rolling direction. (**b**) Strething in transverse direction.

Figure 8 shows the experimental flow stress–strain data of a hot-rolled magnesium alloy sheet measured via uniaxial tensile tests in different directions, and the corresponding results reproduced by different models. It can be seen that they can all be well reproduced, using a numerical algorithm, through the new model. The regression results of the new model are significantly better than those of the power function.

### 4.2. The Effect of the Regression Results of Different Models on the Yielding Property

For anisotropic materials, in order to obtain accurate FE simulation results of plastic deformation, the yielding property of the material should be accurately reproduced via the plastic constitutive model used in the FE analysis. Therefore, the effects of the regression results of different models on the yielding and plastic flow properties were analyzed through the experimental results of a 0.7 mm thick cold-rolled IF steel sheet.

#### 4.2.1. Effect of Regression Accuracy on the Yield Loci

First, the initial yield points of different stress states were determined according to the equivalent strain calculated through the Mises function, which is 0.002. Assuming that the material is incompressible, the initial yield condition can be expressed as

(20) $$\varepsilon_{i}=\frac{2}{\sqrt{3}}\sqrt{\varepsilon_{x}^{2}+\varepsilon_{x}\varepsilon_{y}+\varepsilon_{y}^{2}}=0.002$$

where $\varepsilon_{x},\varepsilon_{y}$ are the true strains along the directions x and y, respectively.

Further, experimental points on the subsequent yield loci can be obtained according to $d^{2}w^{p}=C$, i.e.,

(21) $$\left(\sigma_{x}^{\left(i\right)}-\sigma_{x}^{\left(i-1\right)}\right)\left[f_{x}^{-1}\left(\sigma_{x}^{\left(i\right)}\right)-f_{x}^{-1}\left(\sigma_{x}^{\left(i-1\right)}\right)\right]+\left(\sigma_{y}^{\left(i\right)}-\sigma_{y}^{\left(i-1\right)}\right)\left[f_{y}^{-1}\left(\sigma_{y}^{\left(i\right)}\right)-f_{y}^{-1}\left(\sigma_{y}^{\left(i-1\right)}\right)\right]=d\sigma_{0}^{i}d\varepsilon_{0}^{i}$$

where $\sigma_{x}^{\left(i\right)}$ and $\sigma_{x}^{\left(i-1\right)}$ are the true stress for the steps i and i-1 along the direction *x*, $\sigma_{y}^{\left(i\right)}$ and $\sigma_{y}^{\left(i-1\right)}$ are the true stress for the steps i and i-1 along the direction *y*, $f_{x}^{-1}\left(\sigma_{x}^{\left(i\right)}\right)$ and $f_{x}^{-1}\left(\sigma_{x}^{\left(i-1\right)}\right)$ are the true strains for the steps i and i-1 along the direction *x*, and $f_{y}^{-1}\left(\sigma_{y}^{\left(i\right)}\right)$ and $f_{y}^{-1}\left(\sigma_{y}^{\left(i-1\right)}\right)$ are the true strains for the steps i and i-1 along the direction *y*. The expression of $f_{x\;or\;y}^{-1}\left(\sigma \right)$ see Equation (3), $d\sigma_{0}^{i}$, and $d\varepsilon_{0}^{i}$ are the stress increment and the strain increment from uniaxial tensile testing, respectively.

**Figure 9:**
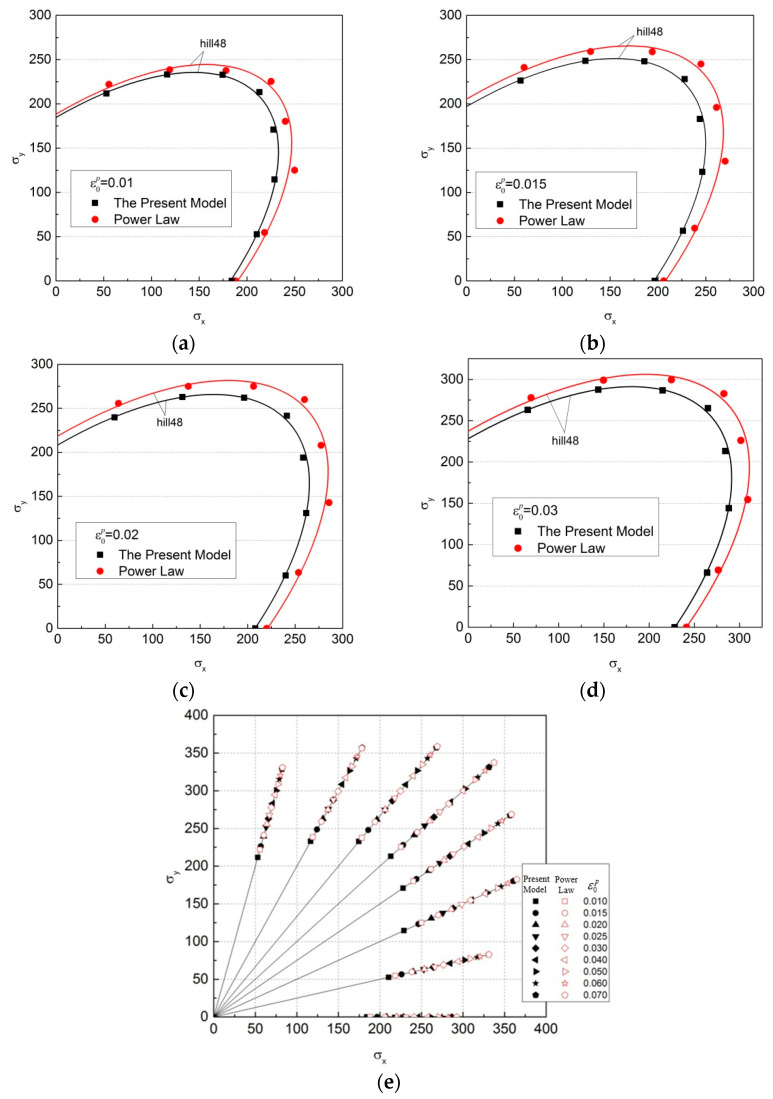
Yield loci of the cold-rolled IF steel sheet which were obtained with the regression results of the new model and the power function ($\varepsilon_{0}^{p}$ is the plastic strain of the uniaxial tensile test). (**a**) $\varepsilon_{0}^{p}=0.01$; (**b**) $\varepsilon_{0}^{p}=0.015$; (**c**) $\varepsilon_{0}^{p}=0.02$; (**d**) $\varepsilon_{0}^{p}=0.03$; (**e**) Yield loci corresponding to different values of $\varepsilon_{0}^{p}$.

Figure 9 shows the yield loci of the cold-rolled IF steel sheet, which were obtained with the regression results of the new model and the power function, respectively. Figure 9a–d are the yield loci corresponding to $\varepsilon_{0}^{p}=0.01,\;0.015,\;0.02$ and 0.03, respectively. The yield loci are obtained via the least-squares method based on the Hill48′s quadratic yield function [36]:

(22) $$f\left(\sigma_{ij}\right)=A\sigma_{x}^{2}+\sigma_{y}^{2}+\bar{B}\sigma_{x}\sigma_{y}+D\tau_{xy}^{2}=\bar{C}$$

where $f(\sigma_{ij})$ represents the yield function of the material element, and $A,\bar{B},\bar{C},D$ are the pending coefficients, which are determined using the experimental data.

**Figure 10:**
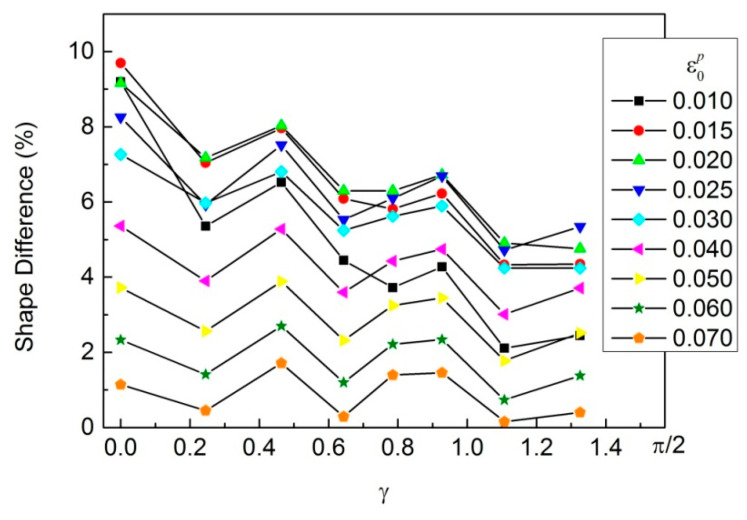
Shape difference in the yield loci obtained based on the regression results of the new model and the power function.

It can be seen that there are significant differences in the yield loci of the cold-rolled IF steel sheet obtained based on the regression results of different models. The regression results have a significant impact on the yield loci of the cold-rolled IF steel sheet. The yield loci of the cold-rolled IF steel sheet obtained based on the new model are significantly smaller than those obtained based on the power function. The smaller the degree of deformation is, the greater the difference in the yield loci will be. The biggest difference between the two shapes is nearly 10%, as shown in Figure 10.

#### 4.2.2. Effect of the Yield Loci on the Predicted Plastic Flow Direction

**Figure 11:**
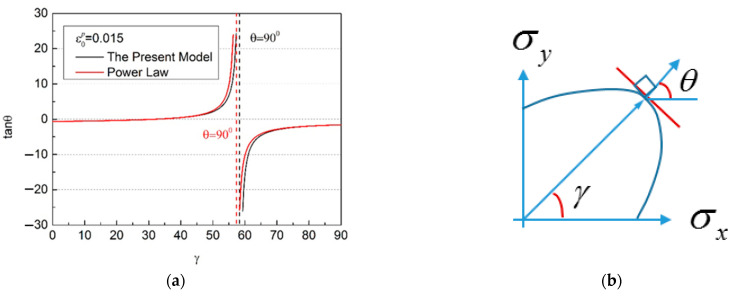
Predicted plastic flow direction in terms of the yield loci shown in Figure 9b. (**a**) Different models can have an impact on regression results; (**b**) Direction of flow.

If the associated flow rule is adopted, then the flow direction of the material will be predicted by the yield function. As mentioned above, the regression results of different models have a significant impact on the yield loci, so will inevitably have a significant impact on the calculated flow direction, as shown in Figure 11. The regression results of different models directly affect the yield loci and plastic flow directions predicted using theory. Higher theoretical prediction accuracy for the yielding and plastic flow properties can be obtained based on a better regression result.

### 4.3. The Effect of the Regression Results of Different Models on the Plastic Flow Property

The influence of the regression results of different models on the plastic flow properties was also analyzed through the experimental results of 0.7 mm thick cold-rolled IF steel sheets. The plastic flow properties of thin-walled metal materials are usually quantitatively described using the ratio of the two in-plane strain increments [7].

The real-time ratio of the two in-plane strain increments in the plastic deformation process can be obtained based on the regression results of the experimental flow stress–strain data of the two directions x and y. Assuming that thin-walled metal materials are uncompressible bodies, the ratio of the two in-plane strain increments can be written as

(23) $$\beta =\frac{\Delta \varepsilon_{x}}{\Delta \varepsilon_{y}}$$

where △*ε_x_* and △*ε_y_* are the strain increments in the directions *x* and *y*, respectively.

Based on Equation (4), the strain increments can be determined using the corresponding stress increments:

(24) $$\left\{\begin{array}{l} \Delta \varepsilon_{x}=\frac{{\left(b_{1x}+\varepsilon_{x}\right)}^{2}}{a_{1x}\varepsilon_{x}{}^{2}+2a_{1x}b_{1x}\varepsilon_{x}+a_{2x}b_{1x}-a_{3x}}\Delta \sigma_{x}\\ \Delta \varepsilon_{y}=\frac{{\left(b_{1y}+\varepsilon_{y}\right)}^{2}}{a_{1y}\varepsilon_{y}^{2}+2a_{1y}b_{1y}\varepsilon_{y}+a_{2y}b_{1y}-a_{3y}}\Delta \sigma_{y} \end{array}\right.$$

where *a_ix_*, *b_jx_* and *a_iy_*, *b_jy_* (*i* = 1, 2, 3, *j* = 1) are the pending coefficients in the directions *x* and *y*, respectively.

By substituting Equation (24) into Equation (23), the real-time ratio of the two in-plane strain increments can be represented by the regression functions in two directions. For comparison purposes, the strain increments in the directions x and y are also determined based on the following power functions:

(25) $$\Delta \varepsilon_{x}=\frac{1}{n_{x}K_{x}^{1/n_{x}}}\sigma_{x}^{\left(1-n_{x}\right)/n_{x}}\Delta \sigma_{x},\;\Delta \varepsilon_{y}=\frac{1}{n_{y}K_{y}^{1/n_{y}}}\sigma_{y}^{\left(1-n_{y}\right)/n_{y}}\Delta \sigma_{y}$$

where $K_{x}$, $K_{y}$ and $n_{x},n_{y}$, are the pending parameters of the power functions in the directions *x* and *y*.

**Figure 12:**
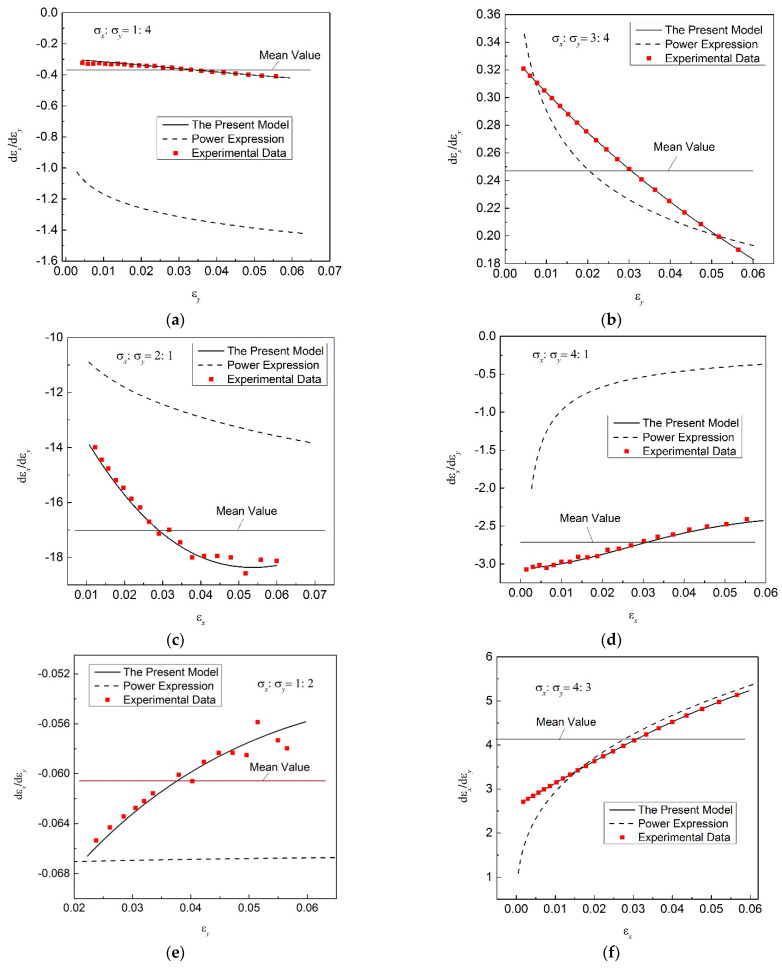
Changeable ratio of the two in-plane strain increments with a progressive hardening process. (**a**) $\sigma_{x}:\sigma_{y}=1:4$; (**b**) $\sigma_{x}:\sigma_{y}=3:4$; (**c**) $\sigma_{x}:\sigma_{y}=2:1$; (**d**) $\sigma_{x}:\sigma_{y}=4:1$; (**e**) $\sigma_{x}:\sigma_{y}=1:2$; (**f**) $\sigma_{x}:\sigma_{y}=4:3$.

Figure 12 shows that the ratio of the two in-plane strain increments varies with the strain value in the tensile direction in real time. The experimental data of *β*-value are also provided in Figure 12.

In Figure 12, there is a big difference in the ratios *β* obtained based on the regression results of different models, and the ratios *β* calculated on the basis of the regression results of the new model are closer to the experimental results. But the *β*-values obtained based on the power function are far from the experimental results, which will lead to a large error in the description of the plastic flow property of the material. In addition, the ratio *β* varies with the hardening process, which is similar to the results in much of the literature [37]. But the variation in the ratio *β* with the hardening process is also different for different loading paths. The ratio *β* increases with the hardening process for the stress ratios *α* = 1:4, 2:1 and 3:4, while in contrast, the ratio *β* decreases with the hardening process for *α* = 4:1, 1:2 and 4:3. This means that a poor predicting accuracy will be obtained if a constant *β*-value is used in the plastic constitutive model of FE simulations, like the value currently used in stamping simulations. An accurate regression model is crucial for describing the plastic flow properties of thin-walled metal materials.

## 5. Conclusions

In this paper, a new regression model was proposed, and the analytical and numerical methods for determining the pending parameters of the new model were introduced. Then, the experimental flow stress–strain data of three kinds of thin-walled metal materials with obvious anisotropy were used to analyze the regression feasibility and universality of the new model. The regression results of other models were also analyzed for comparison. Finally, the effects of the regression results of different models on the yielding and plastic flow properties were analyzed through the experimental results of 0.7 mm thick cold-rolled IF steel sheets; the following conclusions were obtained.

1. The new regression model has a general expression, the regression results of which are all good, except for several combinations of m and n. The combination of n = 2, m = 1 is first recommended after comprehensive balancing of regression accuracy, due to the monotonicity of the first derivative, whether it can be expressed as an explicit function of stress and strain and calculation efficiency.
2. The recommended form of the new model only has four pending coefficients, and the highest-order term in the recommended form of the new model is quadratic, so the functional relationships between stress–strain components can be organized into explicit expressions using this model, which is easy to use for the determination of the coefficients of plastic constitutive models.
3. The new regression model in the recommended form can well reproduce most of the experimental flow stress–strain data of the three kinds of thin-walled metal materials, whether it is in the uniaxial tensile stress state or in the biaxial stress state.
4. The regression results of different models have a significant impact on the yielding properties of 0.7 mm thick cold-rolled IF steel sheets. The yield loci of the cold-rolled IF steel sheets obtained based on the new model are smaller than those obtained based on the power function. The biggest difference between the two shapes is nearly 10%.
5. The regression results of different models have a significant impact on the plastic flow properties of the material, in the case of 0.7 mm thick cold-rolled IF steel sheets. The ratios *β,* calculated on the basis of the regression results of the new model, are much closer to the experimental results.

## Figures and Tables

**Table 1:** Expressions and corresponding regression coefficients in Figure 1.

Expression	Regression Coefficients
$f\left(\varepsilon \right)=\frac{a_{1}\varepsilon +a_{2}}{\varepsilon +b_{1}}$	a1 = 567.5, a2 = 14.62, b1 = 0.07442
$f\left(\varepsilon \right)=\frac{a_{1}\varepsilon +a_{2}}{\varepsilon^{2}+b_{1}\varepsilon +b_{2}}$	a1 = 4.342 × 10^6^, a2 = 1.118 × 10^5^, b1 = 7652, b2 = 569.4
$f\left(\varepsilon \right)=\frac{a_{1}\varepsilon^{2}+a_{2}\varepsilon +a_{3}}{\varepsilon^{2}+b_{1}\varepsilon +b_{2}}$	a1 = 8.274 × 10^5^, a2 = 8.062 × 10^5^, a3 = 1.817 × 10^4^, b1 = 1650, b1 = 92.82
$f\left(\varepsilon \right)=\frac{a_{1}\varepsilon^{2}+a_{2}\varepsilon +a_{3}}{\varepsilon +b_{1}}$	a1 = 501.1, a2 = 488.6, a3 = 11.01, b1 = 0.05626

**Table 2:** Regression parameters of the new model and power function for uniaxial tensile stress–plastic strain curve.

Parameters	a_1_	a_2_	a_3_	b_1_	K	n
Value	178.3195	444.3161	14.0985	0.0908	534.07	0.2270

**Table 3:** Regression coefficients of the SOF model for uniaxial tensile stress–plastic strain curve.

ε_A_	1/4ε_max_	1/2ε_max_	3/4ε_max_
X1	1.9840 × 10^−5^	1.9840 × 10^−5^	1.9840 × 10^−5^
X2	0.1115	0.1060	0.0915
X3	25.0050	25.0050	25.0050

**Table 4:** Overall error of each model for uniaxial tensile stress–plastic strain curve.

Models		SOF		New	Power Function
	$\varepsilon_{A}=1/4\varepsilon_{{}_{\max}}$	$\varepsilon_{A}=1/2\varepsilon_{{}_{\max}}$	$\varepsilon_{A}=3/4\varepsilon_{{}_{\max}}$		
SSE	164.94	231.27	2297.27	10.74	5.51 × 10^3^
MAPE	23.1419	28.0840	90.6631	5.8448	138.0752

**Table 5:** Regression parameters of the new model and power function for biaxial stress–strain curves with $\sigma_{x}/\sigma_{y}=1:2$.

Parameters	a_1_	a_2_	a_3_	b_1_	K	n
x-direction	244.0390	355.9898	−1.3706	−0.0098	640.10	0.2063
y-direction	501.0553	488.6348	11.0152	0.0563	633.77	0.2055

**Table 6:** Regression coefficients of the SOF model for biaxial stress–strain data of $\sigma_{x}/\sigma_{y}=1:2$.

Direction	$\varepsilon_{A}$	$1/4\varepsilon_{{}_{\max}}$	$1/2\varepsilon_{{}_{\max}}$	$3/4\varepsilon_{{}_{\max}}$
x	X1	6.4412 × 10^−5^	6.4412 × 10^−5^	6.4412 × 10^−5^
	X2	2.7040	2.6581	2.5068
	X3	5.1461 × 10^3^	5.1461 × 10^3^	5.1461 × 10^3^
y	X1	3.5685 × 10^−5^	3.5685 × 10^−5^	3.5685 × 10^−5^
	X2	0.3516	0.3486	0.3226
	X3	277.7778	277.7778	277.7778

**Table 7:** Overall error of biaxial stress–strain curves with $\sigma_{x}/\sigma_{y}=1:2$ along direction y.

Models	SOF			New	Power Function
	$\varepsilon_{A}=1/4\varepsilon_{{}_{\max}}$	$\varepsilon_{A}=1/2\varepsilon_{{}_{\max}}$	$\varepsilon_{A}=3/4\varepsilon_{{}_{\max}}$		
SSE	23.30	22.76	213.07	11.27	40100
MAPE	7.00	6.78	23.98	5.20	167.41

**Table 8:** Regression parameters of the new model and power function for biaxial stress–strain curves with different stress ratios.

Direction	Stress Ratios α	a_1_	a_2_	a_3_	b_1_	K	n
X	4:1	784.7988	390.9712	5.9259	0.0349	615.96	0.2184
	2:1	857.9773	418.6266	5.6976	0.0310	620.15	0.1913
	4:3	405.0933	496.7475	8.1409	0.0436	626.80	0.1899
	1:4	−1210.0	67.6097	−0.1804	−0.0038	171.29	0.1861
	1:2	244.0390	355.9898	−1.3706	−0.0098	640.10	0.2063
	3:4	−228.6659	603.1735	6.0591	0.0368	898.05	0.2632
	1:1	488.6147	480.0639	5.4004	0.0317	687.80	0.2082
	1:0	178.3195	444.3161	14.0985	0.0908	534.07	0.2270
y	4:1	−966.1030	73.3982	−0.2008	−0.0046	136.01	0.1364
	2:1	−389.1089	320.5606	−0.7479	−0.0062	671.22	0.2192
	4:3	−625.3158	612.8292	5.1903	0.0346	960.07	0.2856
	1:4	1829.5	244.1159	0.7918	0.0053	585.93	0.2060
	1:2	501.0553	488.6348	11.0152	0.0563	633.77	0.2055
	3:4	−252.9977	606.3043	13.7247	0.0707	651.01	0.2032
	1:1	488.6147	480.0639	5.4004	0.0317	687.80	0.2082

**Table 9:** SSEs of the new model and power function for biaxial stress–strain curves with different stress ratios.

Direction	Stress Ratios α	New Model SSE1x	Power Function SSE2x	SSE1x/SSE2x
x	4:1	17.54	2.79 × 10^6^	6.29 × 10^−6^
	2:1	16.70	3.27 × 10^6^	5.11 × 10^−6^
	4:3	12.07	2.64 × 10^6^	4.57 × 10^−6^
	1:4	1.61	7.50 × 10^4^	2.15 × 10^−5^
	1:2	8.05	7.38 × 10^5^	1.09 × 10^−5^
	3:4	13.65	2.60 × 10^6^	5.25 × 10^−6^
	1:1	8.35	2.10 × 10^6^	3.98 × 10^−6^
	1:0	10.74	3.19 × 10^6^	3.37 × 10^−6^
y	4:1	2.20	8.4 × 10^4^	2.62 × 10^−5^
	2:1	16.74	8.64 × 10^5^	1.9.4 × 10^−5^
	4:3	9.08	1.90 × 10^6^	4.78 × 10^−6^
	1:4	8.87	1.82 × 10^6^	4.87 × 10^−6^
	1:2	11.27	2.93 × 10^6^	3.85 × 10^−6^
	3:4	19.66	2.46 × 10^6^	7.99 × 10^−6^
	1:1	8.35	2.10 × 10^6^	3.98 × 10^−6^
	1:0	-	-	-

## Data Availability

Data are contained within the article.

## Appendix A

**Table A1:** Regression analysis of the general model shown in Equation (1).

m≥n	n	SSE	RMSE	R-Square	m≤n	m	SSE	RMSE	R-Square
m = 6	6	5.419	0.5487	0.9999	n = 6	6	5.419	0.5487	0.9999
	5	5.364	0.5313	0.9999		5	5.364	0.5313	0.9999
	4	5.363	0.5178	0.9999		4	5.411	0.5201	0.9999
	3	5.345	0.5045	0.9999		3	5.506	0.512	0.9999
	2	5.363	0.4937	0.9999		2	5.066	0.4799	0.9999
	1	4.726	0.4533	0.9999		1	5.35	0.4823	0.9999
	0	5.499	0.4787	0.9999		0	6.434	0.5289	0.9999
m = 5	5	5.366	0.518	0.9999	n = 5	5	5.383	0.5188	0.9999
	4	5.366	0.5055	0.9999		4	5.346	0.5045	0.9999
	3	5.366	0.4939	0.9999		3	5.357	0.4935	0.9999
	2	5.367	0.4831	0.9999		2	5.347	0.4821	0.9999
	1	5.494	0.4784	0.9999		1	5.35	0.4721	0.9999
	0	5.79	0.4813	0.9999		0	6.296	0.5122	0.9999
m = 4	4	5.344	0.4929	0.9999	n = 4	4	5.344	0.4929	0.9999
	3	5.359	0.4827	0.9999		3	5.353	0.4824	0.9999
	2	5.451	0.4766	0.9999		2	5.349	0.4721	0.9999
	1	5.494	0.4688	0.9999		1	5.361	0.4631	0.9999
	0	23.27	0.9461	0.9997		0	6.397	0.5059	0.9999
m = 3	3	5.51	0.4791	0.9999	n = 3	3	5.51	0.4791	0.9999
	2	8.506	0.5833	0.9999		2	5.51	0.4695	0.9999
	1	10.9	0.6476	0.9998		1	6.806	0.5116	0.9999
	0	433.1	4.005	0.9938		0	6.803	0.5115	0.9999
m = 2	2	10.97	0.6496	0.9998	n = 2	2	10.97	0.6496	0.9998
	1	12.24	0.6733	0.9998		1	10.97	0.6375	0.9998
	0	433.1	3.933	0.9938		0	57.72	1.462	0.9992
m = 1	1	12.24	0.6612	0.9998	n = 1	1	12.24	0.6612	0.9998

Note: SSE is the residual sum of squares ($SSE=\sum {\left(\sigma_{theoretical}-\sigma_{\mathrm{experimental}}\right)}^{2}$). The smaller the *SSE* is, the better the regression result will be. *SSE* = 0 is an ideal case, i.e., the regression results are completely coincident with the experimental results. *RMSE* is the root-mean-square error ($RMSE={\left[\sum {\left(\sigma_{theoretical}-\sigma_{\mathrm{experimental}}\right)}^{2}/n_{*}\right]}^{1/2}$, where $n_{*}$ is the number of measurements). The smaller the *RMSE* is, the better the regression result will be. R-square ranges from 0 to 1; the closer to one the R-square is, the better the regression result will be. R-square = 1 is an ideal case, i.e., the regression results are completely coincident with the experimental results.
